# Annual Meeting of the American Dental Association

**Published:** 1874-10

**Authors:** 


### ARTICLE VI.

### *Annual Meeting of American Dental Association.*

The fourteenth annual meeting of the American Dental Association was held in the city of Detroit, commencing August 4th and closing August 7th.

The officers elected for the ensuing year were: President—M. S. Dean, Chicago. Vice-Presidents—Geo. W. Keely, Oxford, Ohio; James S. Knapp, New Orleans. Corresponding Secretary—George L. Field, Detroit. Recording Secretary—C. S. Smith, Springfield, Ill. Treasurer—W. H. Goddard, Louisville, Ky. Executive Committee—G. H. Cushing, L. D. Shepard, Geo. L. Field, H. A. Smith, G. R. Thomas, S. B. Palmer, G. C. Daboll, A. L. Brockway.

The Executive Committee announced the following Standing Committees:

On Physiology—Drs. J. N. McQuillen, E. S. Gaylord, J. I. Walker.

On Pathology—Drs. H. Judd, L. D. Shepard, J. S. Knapp.

On History and Microscopy—Drs. J. Taft, E. D. Swayne, W. H. Jackson.

On Chemistry—Drs. H. A. Smith, S. B. Palmer, J. S. Cassiday.

On Therapeutics—Drs. E. A. Bogue, W. O. Kulp, C. C. Carroll.

On Operative Dentistry—Drs. G. H. Cushing, C. S. Stockton, S. H. McCall.

On Mechanical Dentistry—Drs. J. H. Rehwinkle, J. F. Canine, J. Johnson.

On Dental Education—Drs. G. W. Keely, A. H. Brockway, S. Welchens.

On Etiology—Drs. H. S. Chase, E. C. Hawkhurst, C. S. Smith.

On Prize Essays—Drs. Forbes, G. L. Field, R. B. Donaldson.

Niagara Falls was fixed upon as the place of holding the next annual meeting, commencing on the first Tuesday in August, 1875.

During the discussion of the report on Dental Education the following resolutions were offered and laid over till next meeting. We publish them as indicative of the feeling expressed by the members present on this important question.

*Resolved*, That it is the sense of this Association that no dental student should be graduated by any dental college without at least three years' instruction, including private pupilage and college instruction. The latter should in no case embrace less than two regular full courses.

*Resolved*, That the association suggest to the different colleges of this country to appoint a common examining committee, consisting of five members, three to be a quorum, none of whom shall be in connection with any dental college, whose duty it shall be to examine all applicants for graduation and decide upon the same.

*Resolved*, That this associatoin recommend to all local societies the adoption of rules prohibiting their members from taking students for a less period than three years, or for such time as will complete a three years' pupilage.

Dr Allport gave notice that he would, at the next session of the association, bring in an amendment to the constitution requiring that one of the requisites for membership in the association in any dentist who shall begin practice, from and after this time, shall be that he be a graduate either of a medical or dental college.

We see that by a resolution passed at this meeting that members of the profession or dental societies desiring a copy of the Transactions for 1873, can procure them free of cost by addressing the Treasurer, Dr. Goddard, Louisville, and paying postage.—*Missouri Dental Journal.*

